# The acute effect of maximal sprint exercise on dynamic cerebral autoregulation in healthy young adults

**DOI:** 10.14814/phy2.70550

**Published:** 2025-09-12

**Authors:** M. E. Weston, E. L. Curtin, P. Buys, N. Gildea, M. Egaña

**Affiliations:** ^1^ THRIVE Laboratory, Department of Physiology School of Medicine, Trinity College Dublin Dublin Ireland

**Keywords:** cerebral blood flow, directional sensitivity, supramaximal, transfer function analysis, Wingate anaerobic test

## Abstract

This study investigated the acute effect of maximal sprint exercise on dynamic cerebral autoregulation (dCA). Twenty‐one healthy young adults (22.9 ± 3.0 years) completed a single experimental visit where dCA was assessed at rest and 25 min following an all‐out 30‐s Wingate Anaerobic Test (WAnT). Forced oscillations in mean arterial pressure (MAP) during 5 min of repeated sit‐stands, performed at 0.05 Hz, were used to determine dCA. Middle cerebral artery blood velocity (MCAv) was measured using transcranial Doppler ultrasonography, and beat‐to‐beat blood pressure using finger plethysmography. dCA was analyzed using: (1) transfer function analysis and (2) a novel directional sensitivity metric (ΔMCAv_T_/ΔMAP_T_) for each MAP direction. There were no pre versus post‐WAnT differences in gain (0.73 ± 0.30 vs. 0.69 ± 0.21 cm.s^−1^/mmHg, *p* = 0.49), normalized gain (1.10 ± 0.41 vs. 1.20 ± 0.33%/mmHg, *p* = 0.19), phase (0.98 ± 0.22 vs. 1.05 ± 0.24 rad, *p* = 0.18), or coherence (0.82 ± 0.13 vs. 0.90 ± 0.12, *p* = 0.08). Pre‐exercise, ΔMCAv_T_/ΔMAP_T_ was significantly higher during acute decreases versus increases in MAP (1.05 ± 0.37 vs. 0.87 ± 0.31 cm.s^−1^/mmHg, *p* = 0.034), and this persisted post‐exercise (0.93 ± 0.23 vs. 0.81 ± 0.25 cm.s^−1^/mmHg, *p* = 0.017). ΔMCAv_T_/ΔMAP_T_ in either MAP direction was unaffected by the WAnT (*p* = 0.15). These novel findings suggest that dCA was unaltered 25 min following maximal sprint exercise in healthy young adults, while providing further support for hysteresis in the cerebral pressure‐flow relationship.

## 1 INTRODUCTION

Dynamic cerebral autoregulation (dCA) refers to the ability of the cerebrovasculature to adjust cerebral blood flow (CBF) in response to alterations in mean arterial pressure (MAP). Impaired dCA is associated with many neurological conditions, including stroke (Castro et al., 2018), traumatic brain injury (Sviri et al., 2009), and Alzheimer's disease (Claassen & Zhang, 2011). Despite its clinical and physiological relevance, dCA is a complex process, with various methods and techniques used to analyze it (Brassard et al., 2023, 2024), and most dCA metrics are unrelated to each other (Tzeng et al., 2012). Among them, transfer function analysis (TFA) is a commonly utilized method to analyze the relationship between oscillations in MAP and cerebral blood flow velocity (CBv) in the frequency domain (Claassen et al., 2016; Panerai et al., 2023). However, a limitation of TFA is the assumption that the linear dCA responses are symmetrical, as it does not separately analyze CBF adjustments to increases and decreases in MAP (Brassard et al., 2017, 2023, 2024; Labrecque, Smirl, et al., 2022). To overcome this limitation, recent studies have performed directional sensitivity analyses of dCA to evaluate if CBv alterations respond differently to changes in blood pressure depending on the direction of the change.

In this regard, evidence of directional sensitivity has recently been reported in the cerebral pressure‐flow relationship both at rest (Panerai, Barnes, et al., 2023) and during large oscillations in MAP in healthy young adults, assessed through repeated squat‐stand maneuvers (Abbariki et al., 2022; Brassard et al., 2017; Labrecque et al., 2021; Labrecque, Burma, et al., 2022; Roy et al., 2022), oscillatory lower body negative pressure (OLBNP) (Labrecque et al., 2024), and leg press exercise (Allison et al., 2025). Specifically, the changes in CBv during transient increases in MAP are attenuated compared to acute decreases in MAP, often termed hysteresis (Brassard et al., 2017). In some studies, these observations have been suggested to be frequency‐dependent, present during faster (0.10 Hz), but not slower (0.05 Hz) oscillations in MAP (Abbariki et al., 2022; Labrecque et al., 2021, 2024), although evidence of directional sensitivity during slow (0.05 Hz) oscillations is also apparent using different analytical techniques (Brassard et al., 2017; Panerai et al., 2018). These recent observations of directional sensitivity have led to recommendations that future research consider this important phenomenon in dCA assessment and quantification and adopt multi‐metric approaches to dCA analyses (Brassard et al., 2024; Labrecque, Smirl, et al., 2022). Furthermore, it is currently not known if directional sensitivity dCA metrics are associated with TFA‐derived dCA outcomes.

The Wingate anaerobic test (WAnT), a supramaximal, all‐out 30‐s sprint performed on a cycle ergometer, is widely used for evaluating anaerobic performance across different populations and training effectiveness, particularly in high‐intensity sports. The WAnT induces rapid and marked alterations in systemic and cerebral haemodynamics both during (Curtelin et al., 2018; Labrecque et al., 2020) and following (Curtelin et al., 2018; Labrecque et al., 2020; Rossow et al., 2010; Stuckey et al., 2012) exercise. These dynamic changes could present a unique challenge to dCA, particularly during the recovery period, given that the WAnT has been reported to induce pre‐syncope symptoms even in healthy volunteers (Lacewell et al., 2014; Sieck et al., 2016). Indeed, middle cerebral artery blood velocity (MCAv) has been observed to be lower in the 20 min following a WAnT, both during standing rest and during a head‐up tilt challenge (Sieck et al., 2016), but whether dCA is altered remains unknown. This is an important consideration given that autonomic recovery following a WAnT can take more than an hour (Stuckey et al., 2012). Recent studies that investigated the influence of an acute bout of exercise on dCA reported that dCA is transiently impaired in healthy volunteers 10 min following a bout of resistance exercise (Smail et al., 2023), and for up to 4 h following a bout of either high‐intensity interval or continuous moderate aerobic exercise (Burma, Copeland, Macaulay, Khatra, Wright, & Smirl, 2020); but these findings are limited to TFA‐derived dCA outcomes and are not known following supramaximal exercise.

Accordingly, the aim of the present study was to investigate the acute effects of a WAnT on dCA in healthy young adults, analyzed through a multi‐metric approach. Secondary aims of this study were (1) to investigate the directional sensitivity of the cerebral pressure‐flow relationship during sit‐stand maneuvers, and (2) to explore the relationships between TFA and directional sensitivity dCA metrics. It was hypothesized that (1) dCA would be impaired following a WAnT, (2) directional sensitivity would be observed in the cerebral pressure‐flow relationship, and (3) TFA and directional sensitivity dCA metrics would not be significantly related.

## 2 METHODS

### 2.1 Participants

Twenty‐four healthy and recreationally active participants (22.6 ± 2.9 years, 13 males) were initially recruited for this study from the student community at Trinity College Dublin. Following approval from the Faculty of Health Sciences Research Ethics Committee, Trinity College Dublin, written informed consent was obtained from all participants. Participants were initially screened for the study's exclusion criteria, which included contraindications to maximal exercise, smoking, and any current or previous metabolic, cardiovascular, or cerebrovascular disease. The study was conducted in accordance with the principles outlined by the Declaration of Helsinki, except for registration in a database.

### 2.2 Experimental protocol

Participants completed one preliminary and one experimental visit. Upon arrival to the laboratory, participants confirmed that they had adhered to the pretest instructions, which included arriving at least 2 h postprandial, avoiding vigorous exercise and alcohol for the preceding 24 h, and refraining from caffeine on the day of the visit. During the preliminary visit, participants completed a ramp incremental and verification test to exhaustion to determine their maximal oxygen uptake (V̇O_2max_) and were familiarized with the WAnT protocol and the procedures involved in the dCA data acquisition. The experimental visit was completed at least 48 h, but no more than 2 weeks, after the preliminary visit. It involved completing a WAnT, with dCA measurements taken immediately before and 25 min after the sprint exercise bout. All exercise was performed on the same electronically‐braked cycle ergometer (Lode Exclaibur, Lode, Groningen, The Netherlands).

### 2.3 Preliminary visit

Height and weight were taken to the nearest 0.01 m and 0.1 kg, respectively, using a calibrated scale and stadiometer (Seca, Hamburg, Germany). Participants were familiarized with the cycle ergometer, and the saddle and handlebars were adjusted for comfort. Participants were then fitted with a nose clip and mouthpiece (Hans Rudolph, Shawne, USA) attached to a metabolic cart (Innocor, Innovision A/S, Odense, Denmark), which measured breath‐by‐breath oxygen uptake (V̇O_2_), carbon dioxide production (V̇CO_2_), and minute ventilation (V̇_E_). Prior to all testing sessions, the system was calibrated as per the manufacturer's recommendations.

#### 2.3.1 Ramp incremental exercise

Participants completed a 2 min warm‐up at 10 W on the cycle ergometer, before completing a ramp incremental test to exhaustion at a ramp rate of 20–30 W.min^−1^. Participants were asked to maintain a consistent cadence between 70 and 90 revolutions/min (rpm) throughout the test. Exhaustion was deemed to have occurred when the cadence fell below 70 rpm for five consecutive seconds, despite strong verbal encouragement from the researchers. V̇O_2max_ was verified via a supramaximal verification test performed at 105% of ramp test peak power, until exhaustion, following 10 min of rest (Poole & Jones, 2017).

### 2.4 Experimental visit

Participants arrived at the laboratory and completed >10 min of seated rest whilst they were fitted with the experimental measurements. The assessment of dCA was conducted before and 25 min after completing the WAnT. This time point was selected as it occurs during the timeframe in which impaired autonomic function and baroreflex sensitivity have been reported following a single WAnT (Stuckey et al., 2012).

#### 2.4.1 Dynamic cerebral autoregulation (dCA) assessment

**FIGURE 1:**
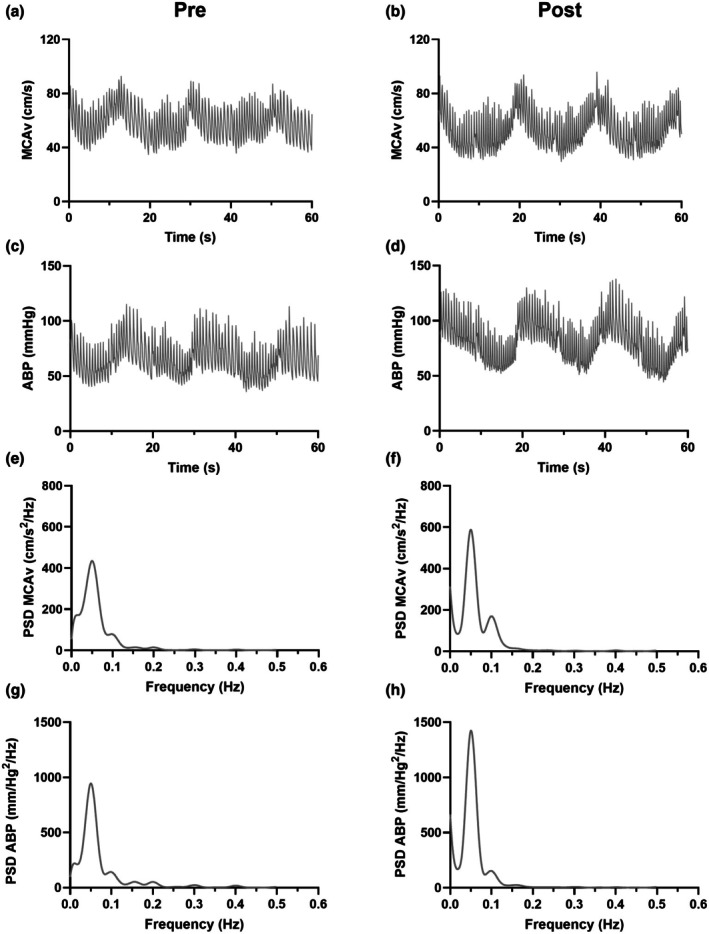
Representative MCAv, ABP data from one participant during 60 s of the repeated sit‐stands at 0.05 Hz before and after the WAnT. ABP, arterial blood pressure; MCAv, middle cerebral artery blood velocity; PSD, power spectrum density.

Following an initial seated baseline of ≥10 mins, participants performed repeated sit‐stands from a chair at a frequency of 0.05 Hz (10‐s sit, 10‐s stand) for 5 min. The chair height was consistent for all participants and resulted in a ~90° knee bend. Forced blood pressure oscillations at this frequency (0.05 Hz) are commonly utilized in the literature (Burma et al., 2024) and represent the very low frequency band, where dCA is considered to have an important influence on the cerebral pressure‐flow relationship (Claassen et al., 2009). An example MCAv and arterial blood pressure (ABP) trace during 60 s of the repeated sit‐stands before and after the WAnT can be seen in Figure 1.

#### 2.4.2 Experimental measures

MCAv was measured bilaterally in all participants using transcranial Doppler ultrasonography. Insonation of the left and right MCA was performed from an initial depth of 45–50 mm using two 2 MHz probes, which were secured in place using a robotic headset (Multigon Industries, Inc., Yonkers, NY). In instances where the MCAv signal could only be located or maintained on one side, unilateral MCAv was used (*n* = 13), with the same side used for pre‐ and post‐exercise measurements within each participant (Weston et al., 2023). Beat‐to‐beat blood pressure was measured at the finger using the arterial volume clamp method (Finometer‐M2, Finapres Medical Systems, The Netherlands), with the hand held at heart level throughout the protocol. Partial pressure of end‐tidal carbon dioxide (P_ET_CO_2_) was continuously measured using a gas analyser (AD Instruments, Colorado) that was calibrated before each visit using gases of known concentration. All data were simultaneously collected at 1000 Hz using an analogue‐to‐digital converter (PowerLab 8/30, AD Instruments, Colorado, USA) and stored for off‐line analysis (LabChart 7, AD Instruments, USA).

#### 2.4.3 Wingate anaerobic test

Participants completed a WAnT on the cycle ergometer with a resistance corresponding to 7.5% of their body weight. Following a 2 min warm‐up at 20 W, participants were given a countdown and instructed to pedal as fast as possible for 30 s while maintaining a seated position, with strong verbal encouragement throughout the protocol. Following the all‐out sprint, participants completed 2 min of unloaded cycling, before a 25 min seated recovery under supervision prior to repeating the dCA assessment.

### 2.5 Data analyses

Beat‐to‐beat mean MCAv and MAP data were exported. Baseline data were taken as the last 60 s of seated rest before completing the repeated sit‐stands. Baseline cerebrovascular conductance index (CVCi) was calculated as: baseline MCAv ÷ baseline MAP. dCA was analyzed using two different methods: (1) transfer function analysis (TFA) and (2) directional sensitivity analysis. P_ET_CO_2_ was averaged across the 5 min of sit‐stands before and after the WAnT. In addition, changes in P_ET_CO_2_ during the sit‐stands was calculated as the difference between the average of the first five breaths compared to the last five breaths (Roy et al., 2022).

#### 2.5.1 Transfer function analysis

Spectral density and transfer function analysis of MAP (input) and MCAv (output) were used to assess dCA using specialised software (CardioBrain 1.1.0) (Pecanha et al., 2024) developed following the latest CARNET guidelines (Panerai et al., 2023). Specifically, beat‐to‐beat data were linearly interpolated and resampled at 10 Hz with a segment size of 1024 points for analysis based on the Welch algorithm. Data were subdivided into four successive windows that overlapped by 50%, and passed through a Hanning window to minimize spectral leakage. TFA gain, normalized gain (%/mmHg), phase, and coherence were sampled at the point estimate of the forced frequency oscillations (0.05 Hz), verified by inspection of the power spectral densities (see Figure 1) (Smirl et al., 2015). Participants were excluded from analyses if coherence dropped below the critical threshold before or after exercise (threshold = 0.41 for 4 windows at 95% significance level), and there was no evidence of phase wrap‐around at any point estimate (Panerai et al., 2023).

#### 2.5.2 Directional sensitivity

A time‐adjusted linear approach was utilized to analyze the directional sensitivity of dCA during the repeated sit‐stands (Labrecque et al., 2021). Absolute changes in MCAv (ΔMCAv) and MAP (ΔMAP) were calculated using the maxima and minima from each sit and stand, respectively. The corresponding time interval (_T_, s) for each increase and decrease in MCAv and MAP was also extracted. Changes in MCAv and MAP were then divided by their corresponding time intervals (ΔMCAv_T_ and ΔMAP_T_, respectively). The time‐adjusted change in MCAv relative to the time‐adjusted change in MAP (ΔMCAv_T_/ΔMAP_T_) was used to quantify dCA, separately for each sit and stand.

**FIGURE 2:**
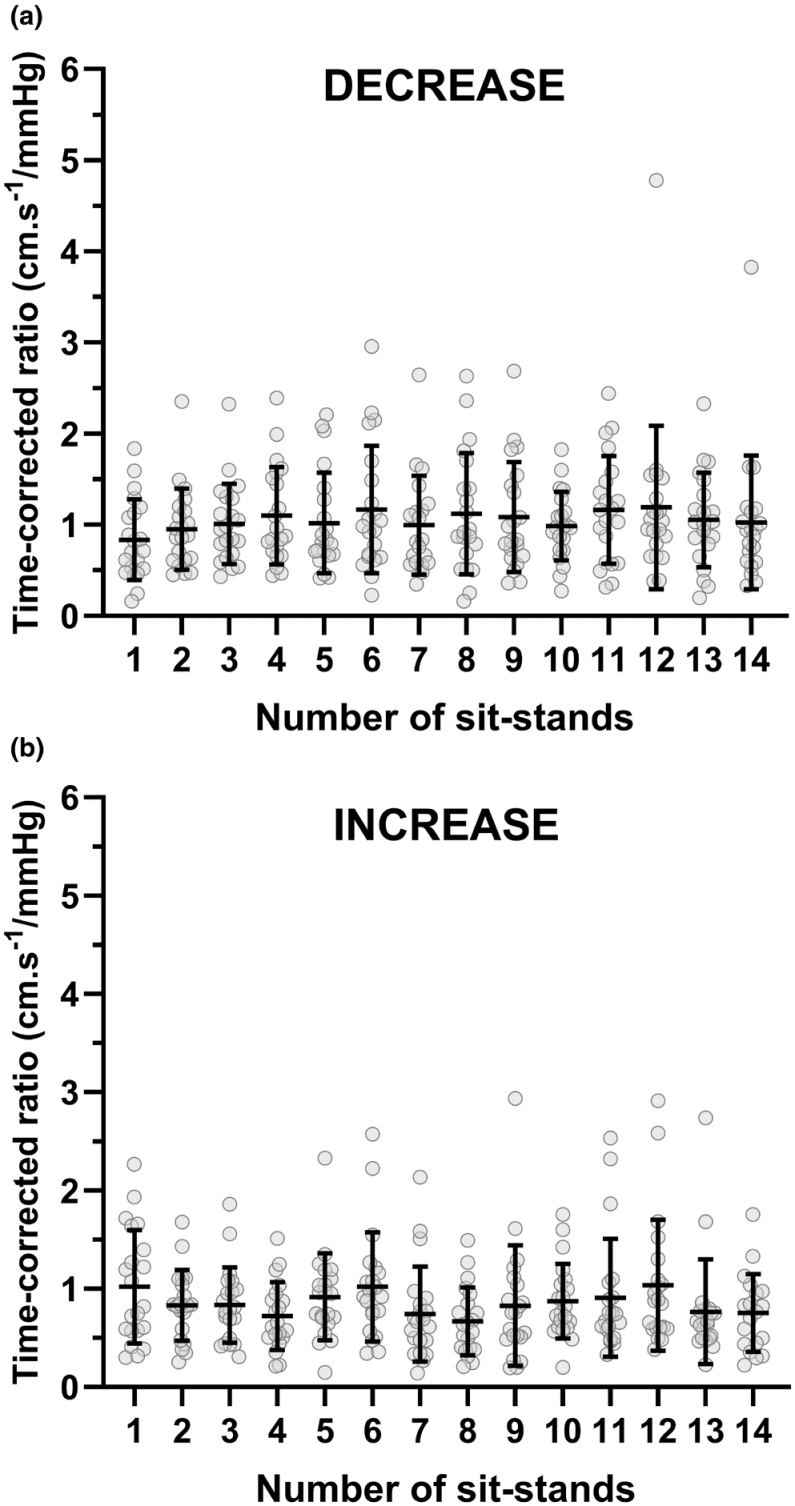
Individual ΔMCAv_T_/ΔMAP_T_ for each sit‐stand repetition during acute decreases (a) and increases (b) in MAP. Numbers on the x‐axis represent squat‐stand repetitions. *n* = 21.

Prior to averaging the data across the 5 min of oscillations, the influence of time was first explored on the individual ΔMCAv_T_/ΔMAP_T_ during each sit‐stand repetition for increases and decreases in MAP (Figure 2). Given that some of these data were not normally distributed, a nonparametric repeated‐measures Friedman test demonstrated that time did not significantly influence the ΔMCAv_T_/ΔMAP_T_ response to increases or decreases in MAP (both *p* > 0.07). Therefore, all variables were then averaged across the 5 min recording for all sits and stands separately (i.e., across 15 sits and 15 stands).

### 2.6 Statistical analyses

This study was powered to detect a moderate effect size (*d* = 0.65) for pre‐post exercise changes in TFA‐derived dCA outcomes, as previously reported for TFA gain and normalized gain following acute resistance exercise (Smail et al., 2023). At an *α* of 0.05 and a power of 0.8, 21 participants were needed, with 24 recruited to account for potential drop‐out or difficulty obtaining and maintaining robust CBv and blood pressure traces.

All data are presented as mean ± SD. Statistical analyses were performed using SPSS version 28 (IBM, Armonk, NY). Statistical significance was set a priori at an α‐level of 0.05. The normality of distributions was checked using the Shapiro–Wilk test. With the exception of TFA coherence, all data were normally distributed.

Prior to exploring the effect of the WAnT on dCA, preliminary analyses were performed to investigate if there was an effect of sex on any dCA outcomes. A 2‐way mixed model ANOVA was performed to explore the effect of time (pre‐ vs. post‐WAnT) by sex on baseline and TFA outcomes. The effect of sex on ΔMCAv_T_/ΔMAP_T_ was explored by a 3‐way mixed model ANOVA (sex × time × direction [decreases vs. increases in MAP]). Effect sizes were calculated and are displayed as partial eta squared, $\eta_{p}^{2}$, and interpreted as: <0.06 = small, 0.06–0.14= moderate, and ≥0.14 = large for ANOVA main and interaction effects (Cohen, 1977). There was no significant main effect of sex (all *p* > 0.23, $\eta_{p}^{2}$ ≤ 0.08) or sex × time interaction (all *p* > 0.05, $\eta_{p}^{2}$ ≤ 0.18) on any baseline or TFA outcomes. Furthermore, there was no significant main effect of sex (*p* = 0.638, $\eta_{p}^{2}$ = 0.01), sex × time (*p* = 0.83, $\eta_{p}^{2}$ < 0.01), sex × direction (*p* = 0.69, $\eta_{p}^{2}$ = 0.01), or sex × time × direction interaction (*p* = 0.11, $\eta_{p}^{2}$ = 0.13) on ΔMCAv_T_/ΔMAP_T_. Therefore, all participants have been combined for subsequent analysis on the effect of exercise on dCA, as in other studies investigating the effect of exercise on dCA (Burma, Copeland, Macaulay, Khatra, Wright, & Smirl, 2020; Roy et al., 2022; Smail et al., 2023).

The effect of the WAnT on TFA outcomes was explored by paired samples *t*‐tests for gain, normalized gain, and phase, and by the Wilcoxon signed‐rank test for coherence. The effects of MAP direction and the WAnT on ΔMCAv_T_/ΔMAP_T_ were explored via a 2‐way repeated measures ANOVA (direction [decreases vs. increases in MAP] × time [pre‐ vs. post‐WAnT]). Significant differences from the ANOVA main and interaction effects were located using Fisher's least significant difference post hoc analyses. The relationships between ΔMCAv_T_/ΔMAP_T_ and TFA‐derived dCA outcomes were explored using Pearson's correlation.

## 3 RESULTS

Due to a loss of MCAv signal or a coherence below the acceptable threshold for TFA (Panerai et al., 2023), three participants (1 male) were removed from analyses. Descriptive characteristics as well as physiological and performance values from the ramp incremental test and WAnT for the final sample size (*n* = 21, 12 males) are shown in Table 1.

**TABLE 1:** Participant characteristics and physiological values from the ramp and WAnT tests.

	*n* = 21 (12 males, 9 females)
Age, years	22.9 ± 3.0
Height, cm	1.73 ± 0.08
Body mass, kg	68.9 ± 10.8
V̇O_2max_, L.min^−1^	3.19 ± 0.92
V̇O_2max_, ml.kg^−1^.min^−1^	45.9 ± 8.9
WAnT peak power, W	799 ± 253
WAnT peak power, W.kg ^−1^	11.5 ± 2.5
WAnT mean power, W	519 ± 137
WAnT mean power, W.kg^−1^	7.5 ± 1.2

*Note*: Data are mean ± SD.

Abbreviations: V̇O_2max_, maximal oxygen uptake. WAnT, Wingate anaerobic test.

### 3.1 Baseline data

Baseline MCAv, MAP, P_ET_CO_2_, and heart rate data before and after the WAnT are shown in Table 2. Following the WAnT, baseline MCAv, CVCi, and P_ET_CO_2_ were significantly lower (*p* ≤ 0.003) and baseline heart rate was significantly higher (*p* < 0.001).

**TABLE 2:** Baseline data before and after the WAnT.

	Pre	Post	*p* Value
Baseline MCAv, cm.s^−1^	66.5 ± 10.8	59.9 ± 9.8	**<0.001**
Baseline MAP, mmHg	81.4 ± 12.7	84.4 ± 15.8	0.39
Baseline CVCi, cm.s^−1^/mmHg	0.83 ± 0.16	0.73 ± 0.16	**0.003**
Baseline P_ET_CO_2_, mmHg	37.1 ± 4.2	30.4 ± 2.9	**<0.001**
Baseline HR, bpm	82 ± 12	102 ± 14	**<0.001**

*Note*: Data are mean ± SD. *n* = 21. Bold indicates significant difference (*p* < 0.05).

Abbreviations: CVCi, cerebrovascular conductance index; HR, heart rate; MAP, mean arterial pressure; MCAv, middle cerebral artery blood velocity; P_ET_CO_2_, partial pressure of end‐tidal carbon dioxide.

### 3.2 Transfer function analysis

**FIGURE 3:**
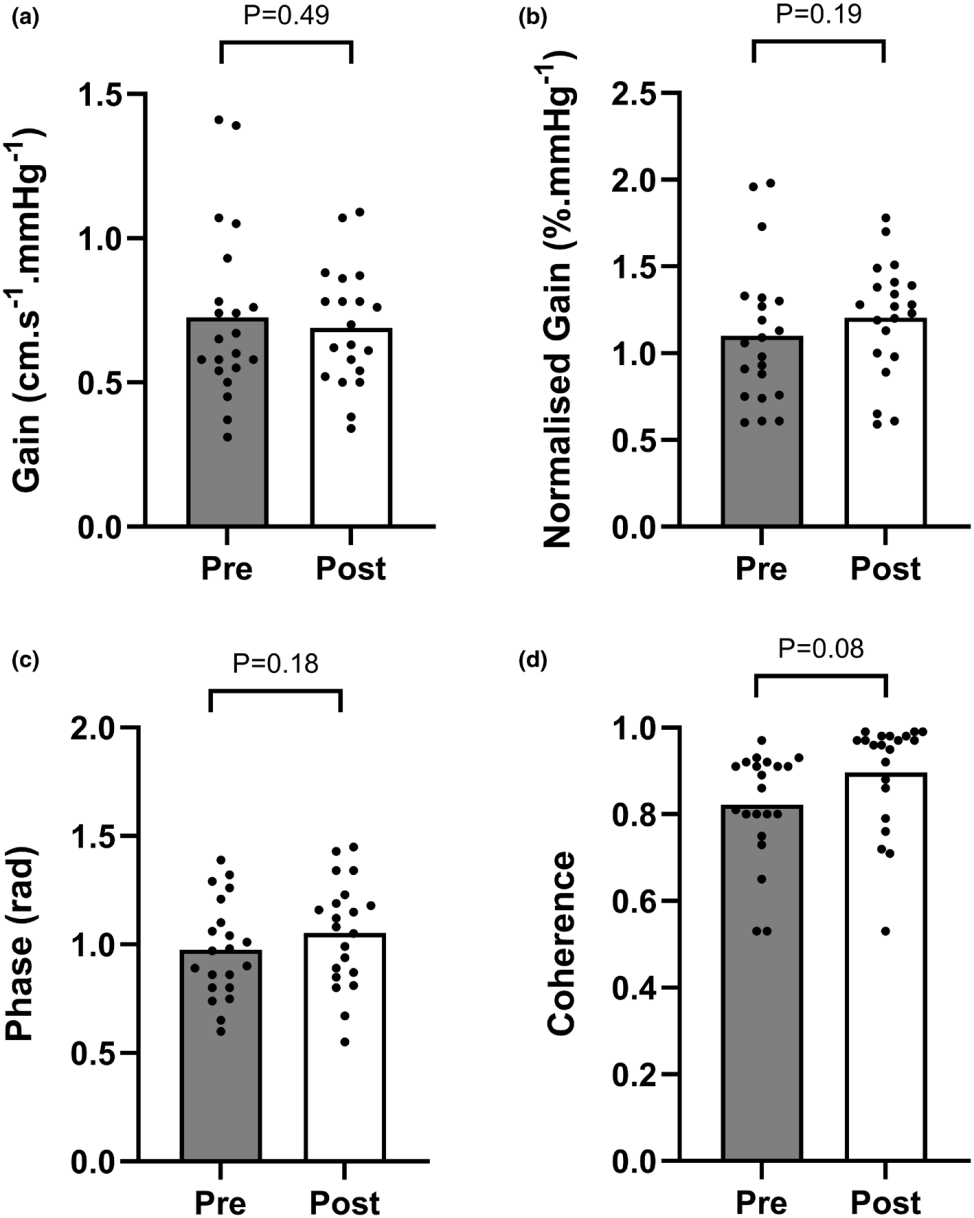
Gain (a), normalized gain (b), phase (c), and coherence (d) from transfer function analysis before (pre) and 25 min following (post) a Wingate anaerobic test. Bars and circles indicate mean and individual data, respectively. *n* = 21.

Figure 3 shows the TFA outcomes before and following the WAnT. There was no significant change in gain (0.73 ± 0.30 vs. 0.69 ± 0.21 cm.s^−1^/mmHg, *p* = 0.49), normalized gain (1.10 ± 0.41 vs. 1.20 ± 0.33%/mmHg, *p* = 0.19), phase (0.98 ± 0.22 vs. 1.05 ± 0.24 rad, *p* = 0.18), or coherence (0.82 ± 0.13 vs. 0.90 ± 0.12, *p* = 0.08). ABP and MCAv power spectrum density estimates at 0.05 Hz before exercise were 438 ± 271 mmHg^2^/Hz and 259 ± 184 cm/s^2^/Hz, respectively, and increased to 1205 ± 1005 mmHg^2^/Hz and 511 ± 431 cm/s^2^/Hz following the WAnT (both *p* < 0.01).

### 3.3 Directional sensitivity

**FIGURE 4:**
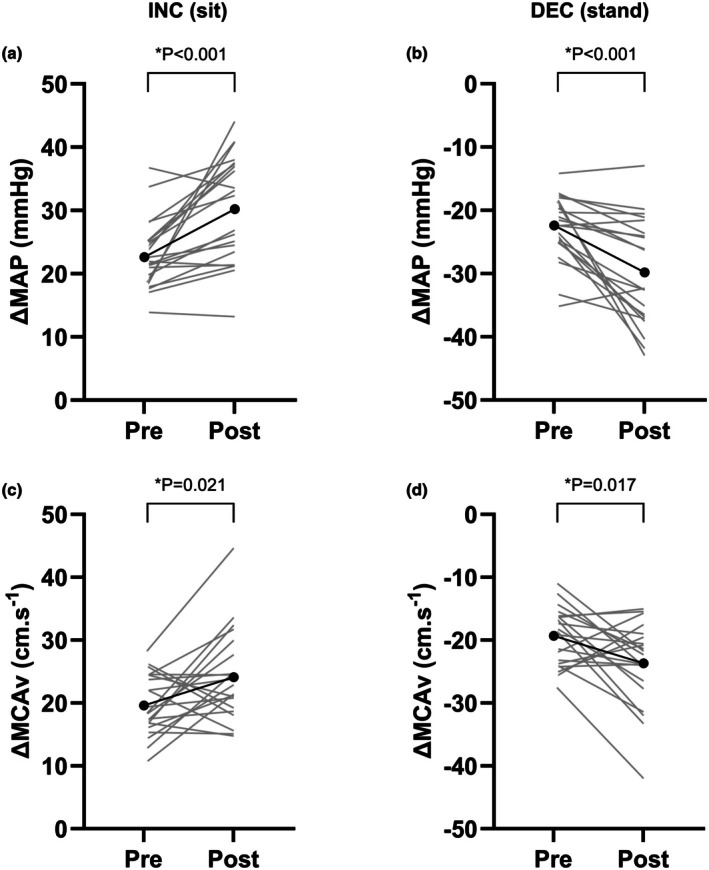
Absolute changes in MAP during acute increases (INC) (a) and decreases (DEC) in MAP (b) and absolute changes in MCAv during acute increases (INC) (c) and decreases (DEC) in MAP (d) before (pre) and 25 min after (post) the Wingate anaerobic test. MAP, mean arterial pressure; MCAv, middle cerebral artery blood velocity. Black and gray lines indicate mean and individual data, respectively. *n* = 21. *p < 0.05.

Figure 4 shows the increases and decreases in MAP and MCAv during sitting and standing, respectively, before and after the WAnT. There was a significant main effect of time on changes in both MAP and MCAv in both directions (*p* ≤ 0.019, $\eta_{p}^{2}$ ≥ 0.26), with significantly greater changes following the WAnT (all *p* ≤ 0.021) (Figure 4). There was also a significant main effect of direction on changes in both MCAv and MAP (*p* = 0.005, $\eta_{p}^{2}$ = 0.35), with significantly greater magnitude of change during MAP increases, compared to decreases. There was no significant time × direction interaction on changes in MCAv (*p* = 0.58, $\eta_{p}^{2}$ = 0.02) or MAP (*p* = 0.26, $\eta_{p}^{2}$ = 0.07).

The time interval for changes in MCAv and MAP showed a significant main effect of direction (*p* < 0.001, $\eta_{p}^{2}$ ≥ 0.68), but there was no effect of time (*p* ≥ 0.11, $\eta_{p}^{2}$ ≤ 0.13) or time × direction interaction (*p* ≥ 0.25, $\eta_{p}^{2}$ ≤ 0.07). Averaged time intervals for MAP and MCAv changes were shorter during MAP increases compared to decreases both before (MAP: 7.7 ± 1.6 vs. 12.3 ± 1.6 s, *p* < 0.001; MCAv: 8.5 ± 1.4 vs. 11.5 ± 1.4 s, *p* < 0.001) and after (MAP: 8.1 ± 1.3 vs. 11.9 ± 1.3 s, *p* < 0.001; MCAv: 8.8 ± 0.9 vs 11.2 ± 1.0 s, *p* < 0.001) exercise.

The rate of change of MAP (MAP_T_) showed a significant main effect of time (*p* = 0.001, $\eta_{p}^{2}$ = 0.41) and direction (*p* < 0.001, $\eta_{p}^{2}$ = 0.79), but no time × direction interaction (*p* = 0.73, $\eta_{p}^{2}$ = 0.01). MAP_T_ was significantly greater during MAP increases compared to MAP decreases both before (3.5 ± 1.1 vs. 1.9 ± 0.5 mmHg/s, *p* < 0.001) and after (4.1 ± 1.1 vs. 2.6 ± 0.8 mmHg/s, *p* < 0.001) the WAnT. Similarly, the rate of change of MCAv (MCAv_T_) showed a significant main effect of time (*p* = 0.026, $\eta_{p}^{2}$ = 0.23) and direction (*p* < 0.001, $\eta_{p}^{2}$ = 0.67), but no time × direction interaction (*p* = 0.80, $\eta_{p}^{2}$ < 0.01). MCAv_T_ was significantly greater during MAP increases compared to MAP decreases both before (2.6 ± 0.8 vs. 1.8 ± 0.5 cm/s^2^, *p* < 0.001) and after (3.0 ± 0.8 vs. 2.2 ± 0.6 cm/s^2^, *p* < 0.001) the WAnT.

**FIGURE 5:**
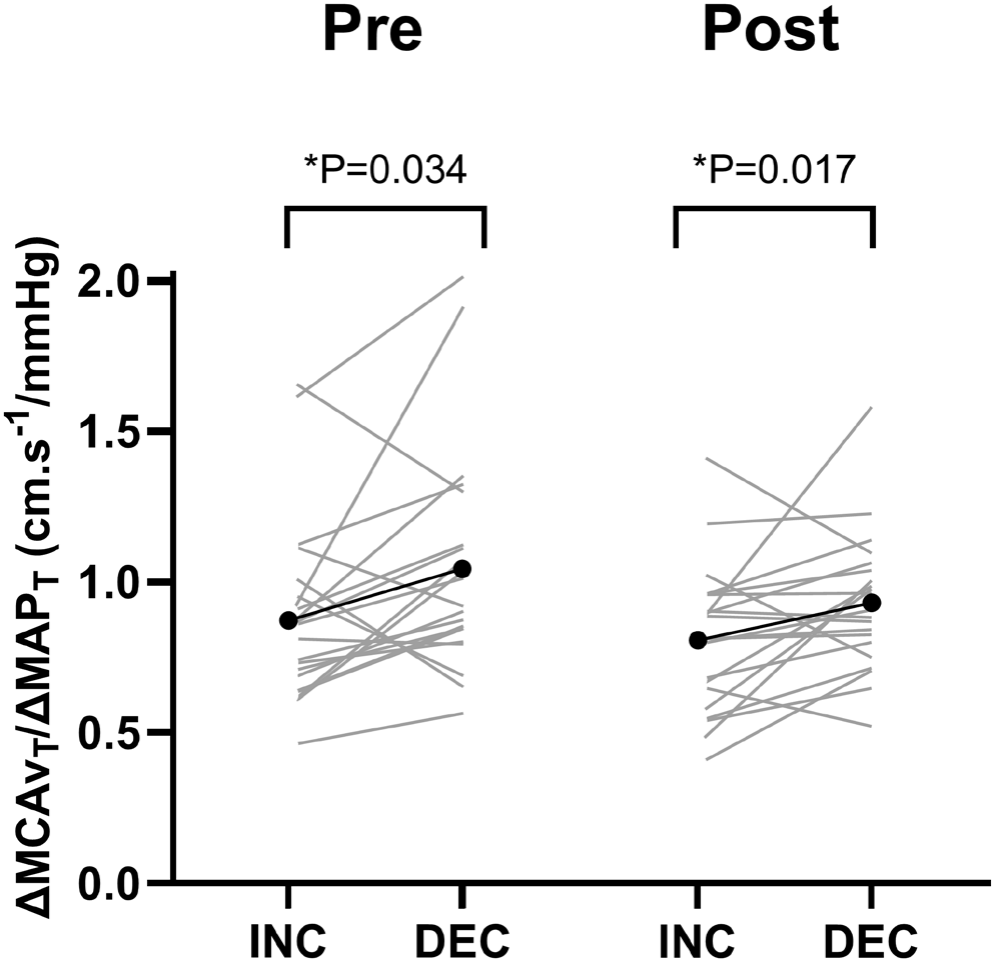
ΔMCAv_T_/ΔMAP_T_ acute increases (INC) and decreases (DEC) during 5 min of repeated sit‐stands before (pre) and 25 min following (post) a Wingate anaerobic test. MAP, mean arterial pressure; MCAv, middle cerebral artery blood velocity. Black and gray lines indicate mean and individual data, respectively. *n* = 21. *p < 0.05.

ΔMCAv_T_/ΔMAP_T_ data before and after the WAnT are shown in Figure 5. There was a significant main effect of direction (*p* = 0.003, $\eta_{p}^{2}$ = 0.37) but no significant effect of time (*p* = 0.15, $\eta_{p}^{2}$ = 0.11) or time × direction interaction (P‐0.76, $\eta_{p}^{2}$ = 0.01). Pre‐exercise, ΔMCAv_T_/ΔMAP_T_ was significantly higher during acute decreases versus increases in MAP (1.05 ± 0.37 vs. 0.87 ± 0.31 cm.s^−1^/mmHg, *p* = 0.034), and this persisted post‐exercise (0.93 ± 0.23 vs. 0.81 ± 0.25 cm.s^−1^/mmHg, *p* = 0.017).

Before the WAnT, 16 participants had a “typical” (lower ΔMCAv_T_/ΔMAP_T_ during transient increases vs. decreases in MAP) response, which was maintained in 13 of these following the WAnT. Four participants had “atypical” (higher ΔMCAv_T_/ΔMAP_T_ during transient increases vs. decreases in MAP) responses pre‐exercise, which were maintained following the WAnT in two of these. The WAnT altered the ΔMCAv_T_/ΔMAP_T_ in six participants.

### 3.4 Changes in P_ET_CO_2_ during the sit‐stands

Averaged P_ET_CO_2_ during the 5 min of sit‐stands was significantly lower following the WAnT (31.2 ± 3.1 mmHg) compared to before the WAnT (37.1 ± 2.9 mmHg, *p* < 0.001). During the sit‐stand protocol, P_ET_CO_2_ did not significantly change before the WAnT (36.8 ± 3.1 to 37.3 ± 3.0 mmHg, *p* = 0.32), but significantly increased during the post‐WAnT sit‐stands (from 30.1 ± 2.9 to 32.2 ± 3.3 mmHg, *p* < 0.001).

### 3.5 Associations between directional sensitivity and TFA‐derived dCA outcomes

ΔMCAv_T_/ΔMAP_T_ during acute increases and decreases in MAP were significantly associated with TFA‐derived gain (*r* = 0.59, *p* = 0.005 and *r* = 0.90, *p* < 0.001, respectively) and normalized gain (*r* = 0.65, *p* = 0.001 and *r* = 0.87, *p* < 0.001, respectively). There were no significant associations between TFA‐derived phase or coherence with ΔMCAv_T_/ΔMAP_T_ in either MAP direction (all *r* < 0.31, *p* > 0.17).

## 4 DISCUSSION

To our knowledge, this is the first study to explore changes in dCA following maximal sprint exercise (WAnT). The main findings of this study were as follows: (1) there was no effect of the WAnT on TFA‐derived dCA outcomes, (2) there was a presence of directional sensitivity in the cerebral pressure‐flow relationship during repeated sit‐stands performed at 0.05 Hz, (3) there was no influence of the WAnT on cerebral pressure‐flow directional sensitivity despite greater changes in MAP during post‐exercise sit‐stands, and (4) directional sensitivity metrics were significantly associated with TFA gain and normalized gain, but not phase or coherence. Collectively, these findings provide further evidence for the directional sensitivity of dCA and suggest that dCA remains unchanged 25 min following maximal sprint exercise in healthy young adults.

### 4.1 Acute effect of Wingate anaerobic exercise on dynamic cerebral autoregulation

The present study observed no significant effect of the WAnT on any of the dCA metrics, either from the TFA or directional sensitivity analyses. In line with previous work, we observed significantly lower MCAv and P_ET_CO_2_ following the WAnT, which have been attributed to exercise‐induced hypocapnia (Sieck et al., 2016). However, 25 mins following the WAnT, resting blood pressure was not altered in the present study, in agreement with previous work (Rossow et al., 2010). However, other studies have reported reductions in diastolic, but not systolic, blood pressure as soon as 15–30 min post a WAnT (Stuckey et al., 2012).

This is the first study to explore changes in dCA following maximal sprint exercise, and despite changes in baseline MCAv, the present study found no effect of the WAnT on dCA. Previous work observed ~13% lower MCAv during a head‐up tilt challenge in the 16 min immediately following a modified WAnT (Sieck et al., 2016), but whether dCA was affected was not investigated. The findings of the present study develop these findings and found that, despite lower CBv, the ability of the cerebrovasculature to buffer acute increases and decreases in MAP was maintained 25 min following maximal sprint exercise. Importantly, this occurred despite greater changes in MAP following exercise, which would provide a greater challenge to dCA post‐WAnT.

The WAnT induces substantial elevations in MAP, heart rate, and cardiac output (Curtelin et al., 2018), and it has been shown that it takes more than an hour for the autonomic system to recover from supramaximal Wingate exercise (Stuckey et al., 2012). Indeed, the significantly elevated resting heart rate 25 min following the WAnT in the present study provides support for increased sympathetic activity following supramaximal exercise. Furthermore, Stuckey et al. (2012) found that baroreflex sensitivity (BRS) is impaired for 1 h following a single WAnT. In this regard, the significantly elevated magnitude of oscillations in MAP during the sit‐stands following the WAnT in the present study may be indicative of poorer BRS, with an attenuated ability to quickly adjust blood pressure during changes in posture. Nevertheless, the present data indicate that dCA had fully recovered 25 min following the WAnT, despite these other cardiovascular alterations, which perhaps indicate faster recovery of dCA. However, it is important to highlight that there were also greater increases and decreases in MCAv during the sit‐stands following exercise. Although dCA appeared to remain intact, these greater changes in both MAP and MCAv during sitting and standing may still have implications for post‐exercise orthostatic tolerance and syncope.

An important consideration in the present study is the significantly lower P_ET_CO_2_ during the post‐WAnT sit‐stands. On average, P_ET_CO_2_ was ~6 mmHg lower during the sit‐stand manoeuvres after exercise. A number of previous studies have used voluntary hyperventilation to induce hypocapnia ranging from −15 mmHg (Aaslid et al., 1989) to −7 mmHg (Edwards et al., 2004) and reported improved dCA metrics in healthy volunteers. Specifically utilizing repeated squat‐stands at 0.05 Hz, dCA phase was significantly improved during hypocapnia in healthy young adults (~11.5 mmHg lower than normocapnia) (Birch et al., 1995). It is therefore possible that the “unchanged” dCA in the present study following the WAnT (when resting P_ET_CO_2_ is lower) is actually indicative of poorer dCA in conditions of resting hypocapnia. However, it has been suggested that the effects of hyperventilation in these previous studies to induce hypocapnia have a more important influence on dCA, rather than PaCO_2_ (Ainslie et al., 2008). Given that the reductions in resting P_ET_CO_2_ in the present study were induced by prior maximal sprint exercise, rather than voluntary hyperventilation during the dCA protocol, the isolated effects of hypocapnia versus the exercise bout are not possible to determine in the present study.

While no other study has investigated the acute effect of maximal sprint exercise on dCA, previous work has observed a transient elevated dCA gain during repeated squat‐stands 10 min following acute resistance exercise (i.e., four sets of 10 repetition back squats), which was found to return to baseline 45 min following exercise (Smail et al., 2023). It is therefore possible that post‐exercise dCA alterations are short‐lived, and that dCA had already recovered by the 25 min time point in the present study, despite previous observations of incomplete autonomic recovery (Stuckey et al., 2012). Indeed, the immediate post‐exercise period is where the greatest risk for syncope occurs following the WAnT (Lacewell et al., 2014; Sieck et al., 2016); therefore, future studies are needed to investigate the time course of dCA changes following maximal sprint exercise. However, altered dCA phase from repeated squat‐stands has been observed for up to 4 h following continuous and high‐intensity interval aerobic exercise (Burma, Copeland, Macaulay, Khatra, Wright, & Smirl, 2020). This suggests that post‐exercise alterations in dCA are complex and dependent upon important factors surrounding the exercise stimulus (intensity, modality, and duration), the dCA analysis method, as well as the time course investigated.

### 4.2 Directional sensitivity

The present study observed directional sensitivity in the cerebral pressure‐flow relationship, with attenuated changes in MCAv during acute increases versus decreases in MAP using repeated sit‐stands performed at 0.05 Hz. Although the presence of directional sensitivity in healthy young adults agrees with previous literature (Abbariki et al., 2022; Brassard et al., 2017; Labrecque et al., 2021, 2024; Labrecque, Burma, et al., 2022; Roy et al., 2022), the present study observed this at a frequency of 0.05 Hz, which supports some studies that have utilized different analytical approaches (Brassard et al., 2017; Panerai et al., 2018) but is in contrast to most of the other recent studies that suggest that directional sensitivity is frequency‐dependent when analyzing ΔMCAv_T_/ΔMAP_T_. Specifically, utilizing repeated squat‐stands, these studies have only found directional sensitivity at 0.10 Hz, with comparable ΔMCAv_T_/ΔMAP_T_ during transient increases and decreases in MAP at 0.05 Hz (Abbariki et al., 2022; Labrecque et al., 2021; Labrecque, Burma, et al., 2022; Roy et al., 2022). A key difference in the methodology of these previous studies compared to the present study is the method used to force oscillations in MAP. To our knowledge, the present study is the first to utilize repeated sit‐stands, as opposed to squat‐stands, to investigate directional sensitivity, which is a commonly utilized protocol to investigate other dCA‐related outcomes (Burma et al., 2024). Sit‐stands were chosen in the present study to ensure safety following supramaximal exercise. The sit‐stands in the present study induced MAP increases and decreases of ~22 mmHg, considerably lower than the ~37 to 47 mmHg reported using squat‐stands (Abbariki et al., 2022; Labrecque et al., 2021; Labrecque, Burma, et al., 2022; Roy et al., 2022). Consequently, the magnitude of the MCAv changes in the present study (~20 cm/s) was also lower than in previous work (30–45 cm/s) (Abbariki et al., 2022; Labrecque et al., 2021; Labrecque, Burma, et al., 2022; Roy et al., 2022).

In addition to the magnitude of the MAP and MCAv changes, sitting versus squatting may also influence the time taken for these adjustments, even when performed for the same duration (10 s each). Consistent with these previous studies, we observed that the averaged time intervals for changes in MAP and MCAv were shorter during transient increases, compared to decreases, in MAP. Our data also demonstrate that the rate of change of MAP was consistently higher during MAP increases compared to decreases. Correspondingly, the rate of change in MCAv also exhibited a similar directional pattern. These findings align with evidence emphasizing that dCA is particularly influenced by the speed at which MAP changes, rather than solely by the amplitude of those changes (Claassen et al., 2021). The greater MAP_T_ during increases likely demands a more rapid cerebrovascular response to maintain cerebral perfusion, which is reflected by increased MCAv_T_, and may be contributing to the presence of directional sensitivity in cerebral autoregulation. When comparing to previous studies utilizing squat‐stands, differences in the magnitude, time, and rate of MAP and MCAv changes from sitting versus squatting may reveal different characteristics of the cerebral pressure‐flow relationship. In addition, squat‐stands present a distinctly more challenging physiological stimulus than sit‐stands, with lower limb muscular contractions resulting in more active engagement of the skeletal muscle pump during squat‐stands (Burma et al., 2024; Tansey et al., 2019), which may also impact the cerebral pressure‐flow relationship. This notion, however, is challenged by recent data using oscillatory lower body negative pressure, inducing MAP changes of ~14 mmHg, which observed directional sensitivity at 0.10 Hz, but not at 0.05 Hz (Labrecque et al., 2024).

The absence of directional sensitivity during MAP oscillations at 0.05 Hz has been suggested to be a result of baroreflex latency, in that the slower changes in MAP during 0.05 Hz oscillations allow sufficient time for cardiovascular‐induced changes to occur via the baroreceptors (Labrecque et al., 2021, 2024). Indeed, baroreflex sensitivity is highest at frequencies of 0.05 Hz and lowest at frequencies of 0.10 Hz (Horsman et al., 2013). However, during sit‐stands at 0.05 Hz, no directional sensitivity of BRS has been observed in young men, which is present at higher frequencies (Horsman et al., 2014); as such, it is unlikely that the findings of this study at baseline are underpinned by BRS involvement.

It is important to note that previous studies have observed directional sensitivity during squat‐stand maneuvers completed at 0.05 Hz using different analytical techniques (Brassard et al., 2017; Panerai et al., 2018), which are in agreement with the present study. In addition, recent literature investigating critical closing pressure and resistance‐area product (Panerai et al., 2025) and utilizing hypercapnia (Panerai et al., 2024) has led to suggestions that the origins of directional sensitivity in dCA are myogenic. These data suggest that vascular smooth muscle is faster to contract than relax, and thus directional sensitivity is an intrinsic characteristic of vascular smooth muscle, which could explain and provide support for the observations of the present study. In support of this notion, it has been suggested that MAP oscillations at 0.05 and 0.10 Hz challenge myogenic and autonomic mechanisms of dCA, respectively (Hamner et al., 2010). Taken collectively, the present study provides further support for the phenomenon of directional sensitivity in healthy adults in the cerebral pressure‐flow relationship during oscillations in MAP, but highlights potential and complex interactions between oscillatory challenge, posture, and frequency, which require further investigation.

The present study is also the first to investigate changes in directional sensitivity subsequent to an acute bout of exercise and found that directional sensitivity was maintained following exercise, with no effect of the WAnT on ΔMCAv_T_/ΔMAP_T_ in either MAP direction. Whether the mechanisms underpinning directional sensitivity are the same before and following exercise is not known and are hard to isolate. In particular, the augmented MCAv response to acute decreases compared to increases in MAP following the WAnT may be impacted by alterations in BRS (Stuckey et al., 2012). An impaired or slower ability of the baroreceptors to make adjustments during acute hypotension would result in greater transmission of MAP reductions to the brain, but whether this is altered by exercise and is sensitive to MAP direction requires investigation. Furthermore, the greater magnitude of MAP oscillations observed post sprint exercise requires greater cerebral vasoconstriction and dilation, which may further emphasize vascular smooth muscle differences in contraction and relaxation rates; thus maintaining the observation of directional sensitivity. Indeed, the WAnT has been shown to acutely reduce arterial compliance following exercise (Rossow et al., 2010), which may result in a poorer ability of the cerebral vessels to dilate in response to transient MAP reductions following exercise. Recent studies investigating chronic exercise models have shown that directional sensitivity may be sensitive to high‐intensity interval training (Abbariki et al., 2022) and habitual exercise training status (Roy et al., 2022). Collectively, these studies highlight the need for future investigations into acute and chronic exercise on directional sensitivity to provide additional insight to TFA‐derived dCA outcomes. Finally, this study is the first to explore the relationships between directional sensitivity and TFA‐derived dCA outcomes and found significant associations between ΔMCAv_T_/ΔMAP_T_ and TFA‐gain and normalized gain, but not phase or coherence. This provides further rationale for future studies to adopt multi‐metric approaches (Brassard et al., 2023, 2024; Labrecque, Smirl, et al., 2022) and supports previous findings in that many dCA outcomes are not related to each other (Tzeng et al., 2012).

### 4.3 Study considerations

A key strength of the present study is the multi‐metric approach to dCA quantification, using directional sensitivity analyses alongside TFA outcomes, providing a more thorough insight into the acute effects of exercise on dCA. This follows the recommendation from a number of contemporary dCA studies (Brassard et al., 2023, 2024; Labrecque, Smirl, et al., 2022). However, the present study is not without its limitations.

Firstly, the present study found no effect of sex on dCA. This is in agreement with previous work utilizing TFA of forced oscillations in MAP in young adults (Burma, Copeland, Macaulay, Khatra, & Smirl, 2020; Smail et al., 2023), although both higher (Labrecque et al., 2019) and lower (Favre & Serrador, 2019) TFA gain have been reported in women, compared to men. Furthermore, menstrual cycle phase was not controlled for in our sample of female participants due to time constraints. While this has been debated (Stanhewicz & Wong, 2020; Wenner & Stachenfeld, 2020), evidence indicates that dCA remains constant across the menstrual cycle (Favre & Serrador, 2019; Johnson et al., 2023). Furthermore, initial evidence suggests that directional sensitivity metrics are not influenced by sex in healthy young adults (Labrecque, Burma, et al., 2022), which is in agreement with the present study. However, recent work has revealed that menstrual cycle phase influences directional sensitivity with greater responses in the mid‐luteal than the early follicular phase, albeit during leg press exercise (Allison et al., 2025). Whether menstrual cycle phase influences the directional sensitivity of dCA following acute exercise is presently not known, and future research exploring this is warranted.

In addition, this study investigated dCA of the MCA only, and therefore these findings cannot be generalized to the posterior cerebral circulation. However, initial evidence suggests that hysteresis in the cerebral pressure–flow relationship is observed in both the MCA and PCA (Labrecque, Burma, et al., 2022). An additional consideration is the analytical approach used to quantify directional sensitivity. Given that no study has compared the current ΔMCAv_T_/ΔMAP_T_ approach with the autoregressive‐moving average model used by Panerai et al. (2018), the utilization of a different approach may have led to different findings.

Furthermore, arterial partial pressure of CO_2_ is a powerful regulator of CBF (Hoiland et al., 2019). This study utilized TCD to measure cerebral blood velocity, which is only an appropriate surrogate of CBF if MCA diameter remains constant (Ainslie & Hoiland, 2014). Changes in MCA diameter are known to occur during marked alterations in P_ET_CO_2_ (+15 mmHg, −13 mmHg) (Ainslie & Hoiland, 2014), so, although resting P_ET_CO_2_ was significantly lower (by ~6 mmHg) following exercise, it is likely that resting MCA diameter was not altered. On the other hand, while P_ET_CO_2_ remained constant during our baseline sit‐stand maneuvers, there was a small but significant increase (+1.3 ± 1.9 mmHg) during the sit‐stands following the WAnT. It should be noted, however, that coherence between MAP and MCAv following exercise was greater than before exercise (*p* = 0.08), providing confidence in the linearity between changes in MAP and MCAv despite the small but significant changes in P_ET_CO_2_ during the post‐exercise protocol. Finally, participants were recreationally active university students, so the findings may not be generalizable to older individuals or those with chronic clinical conditions.

## 5 CONCLUSIONS

The present study found that a single bout of 30 s maximal sprint exercise (WAnT) did not alter any dCA outcomes 25 min following exercise, despite greater changes in MAP during sit‐stands following the exercise bout. The present study also provides further support for directional sensitivity in the cerebral pressure‐flow relationship when utilizing repeated sit‐stands in young active adults. Furthermore, these data challenge the frequency‐dependency notion of this observation, as this was observed at 0.05 Hz in the present study.

## AUTHOR CONTRIBUTIONS

MEW, NG, and ME conceived and designed the research. MEW, ELC, and PB collected data. MEW analyzed the data. All authors edited and revised the manuscript and approved the final version for submission.

## FUNDING INFORMATION

This work was supported by Research Ireland Grant Number: 21/FFP‐P/10097.

## CONFLICT OF INTEREST STATEMENT

The authors declare no conflict of interest.

## ETHICS STATEMENT

This study was approved by the Faculty of Health Sciences Research Ethics Committee (Ref: 230906), Trinity College Dublin. The study was conducted in accordance with the principles outlined by the Declaration of Helsinki, except for registration in a database.

## Data Availability

The data that support the findings of this study are available from the corresponding author upon reasonable request.
